# Oral Hygiene

**Published:** 1900-02

**Authors:** William Oliver

**Affiliations:** Sulphur Springs.


ARTICLE VI.

### ORAL HYGIENE.

DR. WILLIAM OLIVER, SULPHUR SPRINGS.

To understand this subject well, we must first know what it means, then ask ourselves a few questions and find out how the results to be desired may be brought about. Oral hygiene means "the keeping of a clean, sweet and healthy mouth from youth up to old age," also, perhaps, less work withal for the dentist, but it would be more satisfactory to both patient and doctor.

We do not claim nor believe that it would be possible for one to be strict enough, however well drilled he might be on the subject, to prevent sometimes having trouble with his teeth. Hence we proceed to notice some of the most important (as I consider them) points on oral hygiene.

From the time a child begins to "cut its first teeth" its mouth should be looked after by one who is competent to judge of its necessities.

I say this advisedly; if I had not seen children's very first teeth ruined by rubbing through the gums with a thimble or a scalpel, breaking the enamel before they ever saw the light, I would not have mentioned it. This then would be the first point. Don't cut and slash the gums of an infant if it is not absolutely essential, and if it must be done, get a competent person to do it.

Another fruitful cause of diseases of the teeth in childhood is the eating of too much sweets, as candy, etc. For the health of both the body and the teeth the amount of candies should be limited and the little fellow trained to wash his mouth thoroughly with a suitable brush after eating it; also after every meal. If they are trained up this way they will most likely stick to it in after years.

As the years pass on people do things contrary to hygienics and injure the teeth. The human family never having been satisfied with "*means*" has always run to *ex tremes*. We eat food that would actually be more palatable merely warm, as hot as it can be gulped down. The same way with coffee or tea; then we tire of a hot substance and fly to ice tea, ice cream, etc., etc., thereby giving the teeth all the extremes of temperature possible, to say nothing of the abuse of nut-cracking and the eating of acids and condiments, and leaving particles of same on the teeth. Again, we sometimes see a person who never used tobacco (and that and snuff causes the loss of enough teeth), and who keeps his teeth clean and as white as pearl, suffer from the accumulation of tartar on the teeth and the diseases of the gums that attends this condition. This may, and I believe is, often caused by alkaline mineral water.

Another *common* cause of bad teeth is *deformity* of the maxillary bones or of the teeth. The bones failed to attain sufficient dimensions, causing malposition and early decay of the teeth. Of course the remedy and the only hygienic measure is for such a person to visit the dentist regularly and have the offending member drawn out, giving the others room, and make it possible to keep them clean. Last, and most important of all, comes the part the patient cannot do for himself. The most important part the dentist plays in oral hygiene. From some of the many causes always in operation the patient's teeth begin to break down and he seeks the advice of the dentist; and to undertake here to describe the various conditions met with and the remedy for each would be out the province of this paper.

Teeth should be properly filled and cared for so long as we can hope for satisfactory results, but we should not temporize too long, for after all an artificial set of teeth is more hygienic, safer for the patient's health and the dentist's reputation, than any set of natural teeth that persistently break down after being carefully and scientifically filled.—*Texas Dental Journal.*

